# Editorial: Toll-Like Receptors Throughout Life: From Controlling Physiological Processes to Determinants of Disease

**DOI:** 10.3389/fcell.2022.898569

**Published:** 2022-04-04

**Authors:** Francesca Arnaboldi, Silvia Selleri, Michele Sommariva

**Affiliations:** ^1^ Dipartimento di Scienze Biomediche per la Salute, Università degli Studi di Milano, Milan, Italy; ^2^ CHU Sainte-Justine, Montréal, QC, Canada

**Keywords:** toll-like receptor, development, immune system, pathology, physiology

Since their discovery, Toll-like receptors (TLRs) have been deeply investigated in different areas of the biomedical research. TLRs are usually associated to their ability to trigger a potent immune response against invading pathogens (Kawasaki and Kawai, 2014; Vijay, 2018) and the study by Valdés-López et al. is another proof in this scenario. The Authors described the involvement of the TLR4 signaling as important factor in inducing an antiviral response, mediated by macrophages, against Chikungunya Virus. However, the importance of TLRs is not restricted to the detection of potential harmful microorganisms. It should be recalled that all the mucosal surfaces of human body are virtually colonized by bacteria, the so-called microbiota, and TLRs continuously interact with these commensal microbes, in a bidirectional crosstalk aimed at preserving homeostasis (Semin et al., 2021), as reported in the review by Le Noci et al. By sensing pathogens and commensals, TLRs provide a double beneficial effect either protecting the host and, at the same time, maintaining a good health status. However, the activation of TLRs not always produces a positive outcome and the initiation of a inflammatory process, mediated by TLR signaling pathway, can be associated to the pathogenesis of different diseases, including cancer (Cook et al., 2004). For instance, Zheng et al. investigated the role of TLR9 during the transition from acute kidney injury (AKI) to chronic kidney disease (CKD), demonstrating that TLR9 expressed by macrophages has a critical role in this process. Moreover, it has been observed that bacteria present in the tumor microenvironment trigger TLRs expressed by tumor-infiltrating immune cells but, instead of generating an anti-tumor immune response, promote immunosuppression and sustain cancer cell growth. The impact of TLRs on cancer development and progression has been also evaluated considering the prognostic value of these receptors, as shown in the study by Lu et al. The Authors observed a positive correlation between TLR4 expression and the overall survival of bladder cancer patients. Moreover, low TLR4 expression was also associated to a better response to chemotherapy. Since TLRs can be included in the etiology of several pathological conditions, they can consequently represent potential good targets for novel therapeutic options (El-Zayat et al., 2019). For example, decreasing TLR expression by quercetin treatment was able to reduce the mortality of broilers by impacting on intestinal microbiota composition Ying et al. Exploiting their immune-activation ability, TLRs have been also investigated as immunotherapeutic drugs, alone or in combination with other therapeutic regimens, as comprehensively described by Farooq et al.

As previously mentioned, there is no doubt about the involvement of TLRs in immune response. However, it is now clear that TLRs are key players in different biological processes not always strictly related to their well-recognized immune function. For example, in *Drosophila melanogaster* the protein Toll is described to establish the dorsal-ventral embryonic polarity (Valanne et al., 2011). As their fruit fly counterpart, mammalian TLRs also play an active role in embryonic development (Anthoney et al., 2018). Accordingly, Yiu et al. found that TLR5 is extremely important for a correct placental formation that resulted negatively affected by TLR5 deletion.

All the manuscripts briefly presented here represent an excellent example of how the study of TLRs can cover different research interests. Although the seven published papers share a common background, it is clear that these studies cover topics that are very different from each other, as a consequence of the pleiotropic activity exerted by TLRs in different biological contexts.
